# Laparoscopic Management of Ovarian Torsion in the Third Trimester of Pregnancy: A Case Report and Literature Review

**DOI:** 10.1002/ccr3.73266

**Published:** 2026-08-02

**Authors:** Aysel Nalçakan Ergünay, Edis Kahraman, Emine Karabük, Turgut Aydın

**Affiliations:** ^1^ Department of Obstetrics and Gynecology Acıbadem Mehmet Ali Aydınlar University Atakent Hospital Istanbul Turkey

**Keywords:** dermoid cyst, laparoscopy, minimally invasive surgery, ovarian torsion, third‐trimester

## Abstract

Adnexal torsion in pregnancy is an uncommon surgical emergency that may compromise both ovarian viability and pregnancy outcome. Diagnosis in late gestation is particularly challenging because of nonspecific symptoms and displacement of the adnexa. Although laparoscopy is well established in early pregnancy, evidence supporting its use in the third trimester remains limited. We report a case of ovarian torsion occurring at 30 + 1 weeks of gestation that was successfully managed laparoscopically using pregnancy‐adapted techniques. The patient presented with acute abdominal pain, and imaging revealed a right adnexal mass with no detectable intraovarian arterial or venous flow. Emergency laparoscopy was performed within a few hours of admission. Abdominal access was achieved using a 10‐mm optical trocar placed under ultrasound guidance approximately 6 cm above the uterine fundus, and pneumoperitoneum was reduced to 12 mmHg after initial access. Detorsion restored the appearance of ovarian perfusion, and ovarian‐sparing cystectomy was performed for the underlying mature cystic teratoma. Postoperative recovery was uneventful, the pregnancy progressed to spontaneous term delivery, and postpartum ultrasonography demonstrated normal right‐ovarian morphology with preserved vascularization. A focused review identified only a limited number of comparable laparoscopic cases at or beyond 28 weeks of gestation. This case adds to the limited evidence supporting individualized laparoscopic management of ovarian torsion in late pregnancy when pregnancy‐specific precautions and multidisciplinary expertise are available.

## Key Clinical Message

Ovarian torsion may occur even in late pregnancy and is often difficult to diagnose because of nonspecific symptoms and altered anatomy. In hemodynamically stable patients, pregnancy‐adapted laparoscopic management with low‐pressure pneumoperitoneum allows timely detorsion, ovarian preservation, and favorable maternal and neonatal outcomes.

## Graphical abstract

Laparoscopic treatment of right ovarian torsion caused by a mature cystic teratoma at 30 + 1 weeks of gestation. The enlarged, edematous, and cyanotic torsed ovary before detorsion A) demonstrated improved color and turgor after detorsion and ovarian‐sparing cystectomy B), consistent with reperfusion and ovarian preservation.

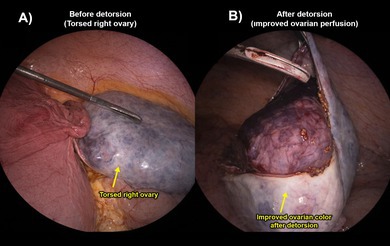

## 1 Introduction

Acute abdominal pain during pregnancy represents a substantial diagnostic challenge because of its nonspecific presentation and broad obstetric and nonobstetric differential diagnosis [1]. Ovarian torsion is uncommon but clinically important, with a population‐based incidence of approximately 1.6 per 10,000 pregnancies [2]. Although most pregnancy‐associated torsions occur during the first trimester, approximately one‐fifth have been reported in the third trimester [1]. The reduced frequency later in gestation is generally attributed to restricted adnexal mobility as the uterus enlarges; nevertheless, torsion may occur throughout pregnancy [1, 3].

An adnexal mass is an important predisposing factor. Benign ovarian lesions, including corpus luteum cysts, mature cystic teratomas (dermoid cysts), and serous or mucinous cystadenomas, are frequently reported in pregnancy‐associated torsion [1, 3]. Delayed intervention may lead to progressive venous and arterial compromise, hemorrhagic infarction, loss of the adnexa, peritonitis, and secondary obstetric complications. Therefore, persistent clinical suspicion warrants prompt operative assessment [1, 3].

Although laparotomy was historically favored in advanced pregnancy, contemporary evidence and guidelines support laparoscopy in any trimester when surgery is clinically indicated and pregnancy‐adapted techniques are used [4, 5, 6, 7, 8]. Published experience with laparoscopic treatment of ovarian or tubo‐ovarian torsion at or beyond 28 weeks remains limited and consists mainly of case reports and small case series [8, 9, 10, 11, 12, 13, 14, 15, 16, 17, 18]. We report a third‐trimester ovarian torsion caused by a mature cystic teratoma that was successfully managed laparoscopically with ovarian preservation and review the literature with a focus on management in advanced gestation.

## 2 Case History/Examination

A 28‐year‐old woman (G2P1) at 30 + 1 weeks of gestation presented directly to our hospital after experiencing progressively worsening right lower quadrant abdominal pain for approximately 3 days. The pain had acutely intensified on the day of admission and was accompanied by nausea and vomiting. Her medical and surgical histories were unremarkable, and the pregnancy had been uncomplicated until the onset of these symptoms.

On initial evaluation, the patient was afebrile and hemodynamically stable. Abdominal examination demonstrated localized tenderness and guarding in the right lower quadrant without rebound tenderness or rigidity. The uterus was appropriate for gestational age, and no uterine contractions were observed.

## 3 Differential Diagnosis, Investigations, and Treatment

Given the clinical presentation, the differential diagnosis included obstetric and nonobstetric causes of acute right lower quadrant pain in pregnancy. The absence of uterine contractions or cervical change, together with reassuring fetal monitoring, made an acute obstetric cause less likely, whereas the known adnexal mass and absent intraovarian arterial and venous flow strongly supported ovarian torsion.

Ten days prior to presentation, magnetic resonance imaging (MRI), performed for intermittent discomfort, had demonstrated an 8‐cm right ovarian dermoid cyst with preserved vascular flow and no evidence of torsion.

**FIGURE 1:**
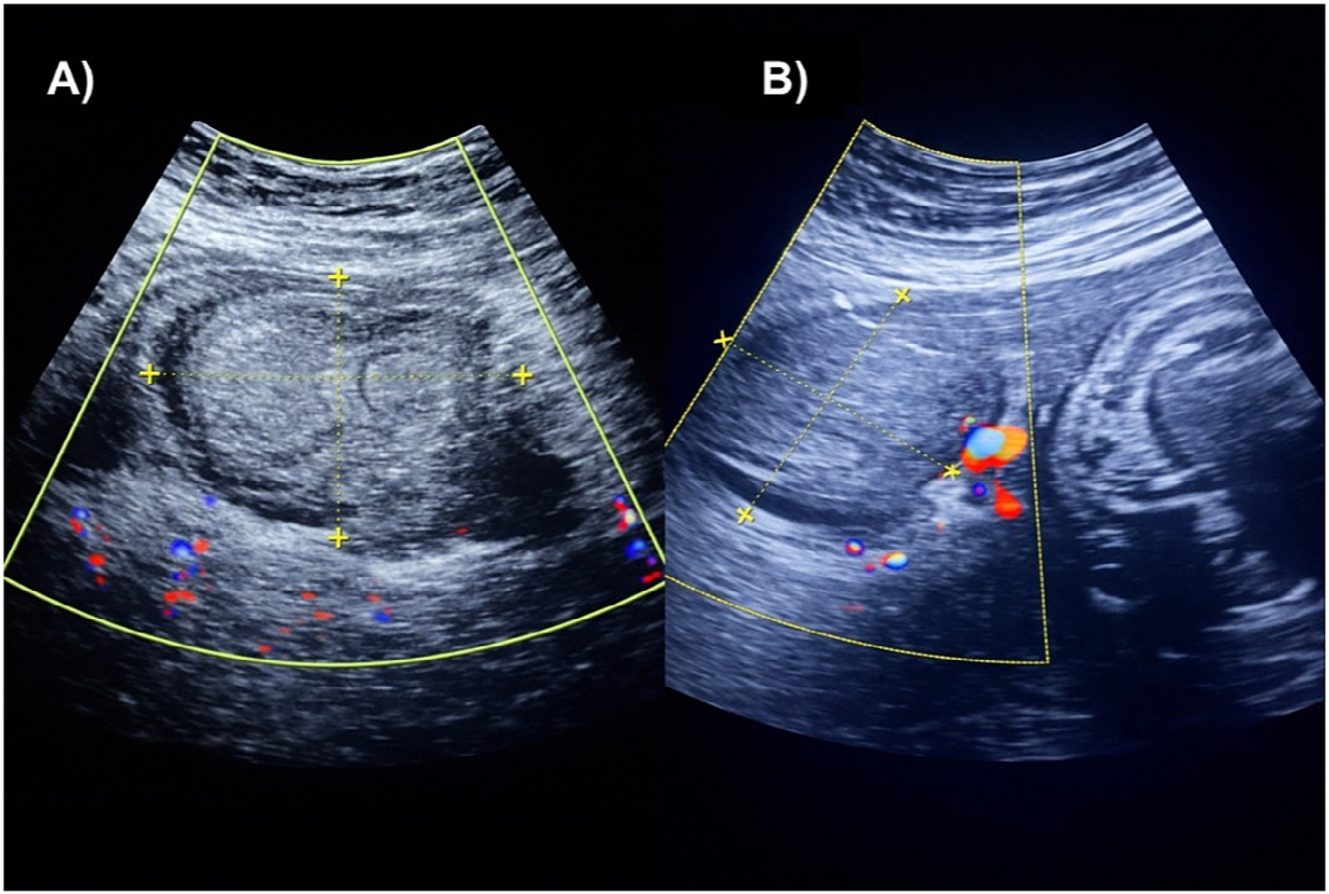
Transabdominal ultrasonography and color Doppler imaging demonstrating an enlarged right adnexal mass with heterogeneous echotexture and impaired vascular flow during late pregnancy. (A) Gray‐scale ultrasonography showing an enlarged, heterogeneous adnexal mass. (B) Color Doppler ultrasonography demonstrating no detectable intralesional vascular flow within the right ovary; adjacent vascular signals remain visible.

At admission, transabdominal ultrasonography confirmed a viable intrauterine pregnancy with a fetal heart rate of 145 beats per minute and normal amniotic fluid volume. A bilocular right adnexal cystic mass measuring approximately 7 × 5 cm and 5 × 4 cm was visualized. Color Doppler imaging demonstrated no detectable arterial or venous flow within the right ovary, while adjacent vascular signals remained visible, raising strong suspicion of ovarian torsion (Figure 1). The cervix was closed, and fetal monitoring showed a reactive tracing without uterine activity. Following prompt clinical and ultrasonographic evaluation, ovarian torsion was strongly suspected. No conservative management was attempted, and emergency laparoscopic intervention was performed within a few hours of admission following multidisciplinary consultation.

The patient was positioned with a left lateral tilt to prevent aortocaval compression, and general anesthesia was administered. Prophylactic intravenous cefazolin was given, and intraoperative mechanical thromboprophylaxis was applied. Abdominal access was achieved using a 10‐mm optical trocar placed under ultrasound guidance approximately 6 cm above the uterine fundus. Pneumoperitoneum was initially established at 15 mmHg to facilitate trocar placement and subsequently reduced to 12 mmHg. Two additional 5‐mm trocars were placed under direct visualization.

**FIGURE 2:**
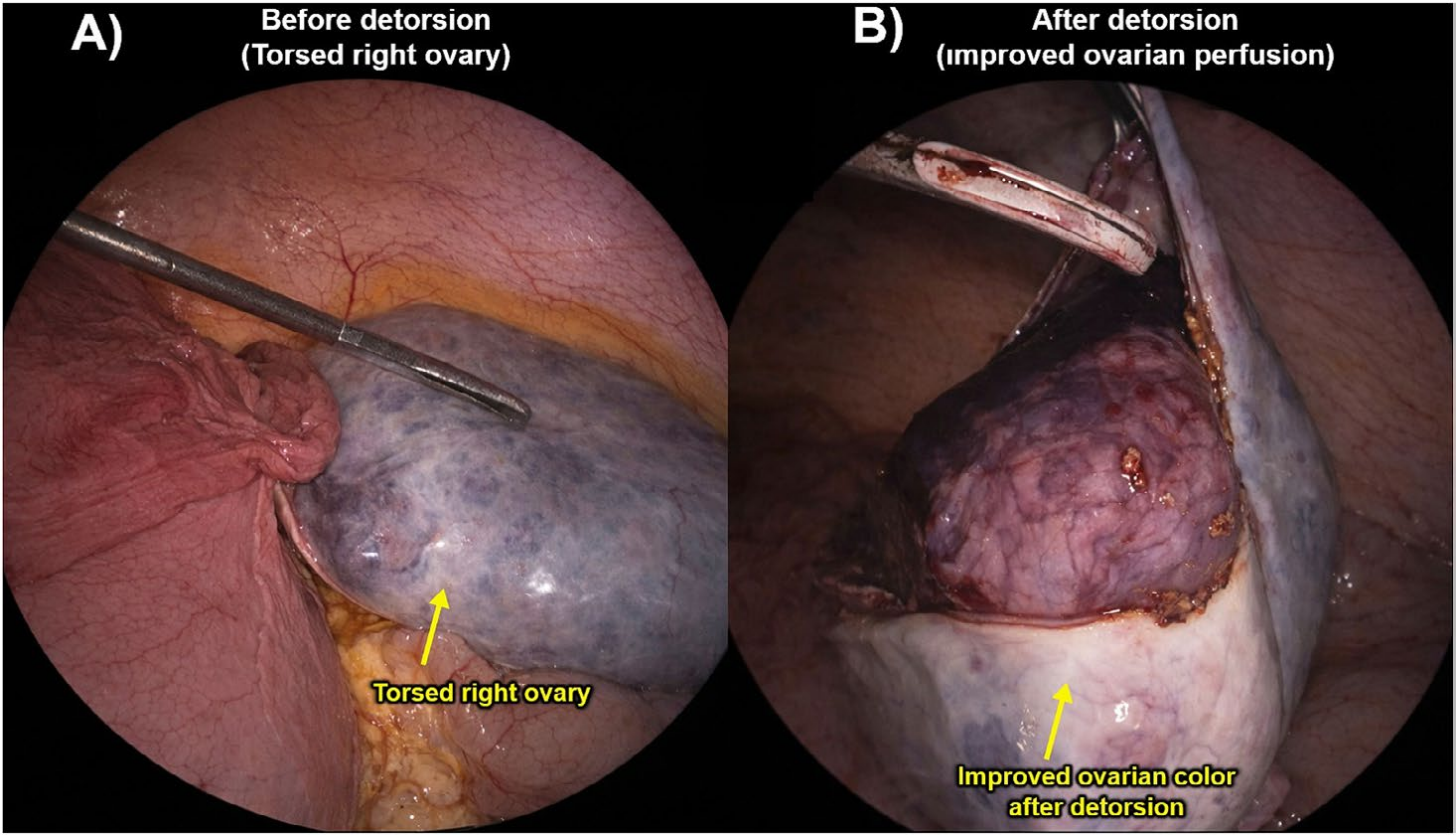
Intraoperative laparoscopic findings before and after detorsion of the right adnexa. (A) Enlarged, edematous, and cyanotic torsed right ovary before detorsion; the arrow identifies the torsed ovary. (B) Appearance of the right ovary after detorsion and cystectomy, showing improved color and turgor consistent with reperfusion; the arrow identifies the preserved ovarian tissue.

Laparoscopic exploration revealed a markedly enlarged, edematous, and cyanotic right ovary containing a multicystic mass, with approximately 720° torsion of the adnexa (Figure 2A). Gentle detorsion was performed using atraumatic graspers, resulting in progressive improvement in ovarian color and turgor within 5 min, consistent with reperfusion (Figure 2B). Given the restoration of blood flow and absence of gross necrosis, ovarian preservation was pursued. Because the dermoid cyst was considered the underlying mechanical factor predisposing the adnexa to torsion, leaving it in situ was thought to maintain the risk of recurrent torsion during the remainder of pregnancy. As ovarian‐sparing cystectomy was judged technically feasible with an acceptable incremental operative risk, it was performed during the same procedure, thereby also avoiding the need for a second operation after delivery. Following controlled decompression of the sebaceous contents, cystectomy was completed using a bipolar vessel‐sealing device, and the specimen was removed in an endoscopic retrieval bag. The operative duration was 42 min, with minimal blood loss and no intraoperative complications.

## 4 Conclusion and Results (Outcome and Follow‐Up)

Postoperatively, maternal recovery was uneventful, and fetal monitoring remained reassuring. The patient was discharged on postoperative day 1. Histopathological examination confirmed a mature cystic teratoma with focal hemorrhagic necrosis consistent with torsion, while the remaining ovarian tissue was viable. The remainder of the pregnancy proceeded without complications. At 40 weeks of gestation, the patient experienced spontaneous onset of labor and delivered a healthy male neonate weighing 3600 g, with Apgar scores of 9 and 10 at 1 and 5 min, respectively. At postpartum follow‐up, ultrasonography demonstrated a right ovary with normal morphology and preserved vascularization. No clinical symptoms or findings suggestive of recurrent adnexal torsion were observed during follow‐up.

This case demonstrates that ovarian torsion caused by a mature cystic teratoma can occur in late pregnancy despite recent imaging showing preserved ovarian perfusion. In appropriately selected patients, prompt laparoscopic detorsion with ovarian‐sparing cystectomy may be successfully performed using pregnancy‐adapted trocar placement, controlled pneumoperitoneum, and multidisciplinary perioperative care. The favorable obstetric course and preserved postpartum ovarian vascularization in this case add to the limited evidence supporting individualized minimally invasive management in advanced gestation.

## 5 Discussion

Adnexal torsion during the third trimester is rare and diagnostically challenging because its symptoms overlap with common obstetric and gastrointestinal conditions, while the enlarged uterus limits physical examination and displaces the adnexa [1, 3]. Doppler ultrasonography may support the diagnosis but is not definitive; preserved flow does not exclude torsion, whereas absent intraovarian flow should be interpreted together with the clinical presentation [1]. In the present case, worsening unilateral pain and no detectable intraovarian arterial or venous flow prompted urgent surgery. The MRI obtained only 10 days earlier had shown preserved ovarian perfusion, illustrating that recent reassuring imaging does not exclude the subsequent development of torsion when symptoms evolve.

Prompt operative assessment is required when torsion is strongly suspected to minimize ischemic injury and preserve ovarian tissue [1, 3]. In this case, detorsion was performed first and produced rapid improvement in ovarian color and turgor. Because the mature cystic teratoma remained a structural risk factor for recurrent torsion, detorsion alone was considered insufficient. Ovarian‐sparing cystectomy was therefore completed during the same operation after reperfusion had been observed. This individualized approach addressed the underlying lesion, preserved viable ovarian tissue, and avoided a planned postpartum operation [19].

Pregnancy‐adapted technique is central to laparoscopy in advanced gestation. Recommended precautions include left lateral displacement to reduce aortocaval compression, placement of the primary and secondary ports according to fundal height, abdominal entry away from the uterus, and use of the lowest insufflation pressure that provides adequate exposure [5, 6, 7]. In the present case, abdominal access was achieved using a 10‐mm optical trocar placed under ultrasound guidance approximately 6 cm above the uterine fundus, and intra‐abdominal pressure was reduced from 15 to 12 mmHg after initial access. These adaptations provided adequate visualization without uterine injury or maternal hemodynamic instability.

Perioperative care requires coordination among obstetric, surgical, and anesthetic teams. Fetal heart activity should be assessed before and after surgery when the fetus is viable [5, 6, 7]. Routine prophylactic tocolysis is not recommended in the absence of uterine contractions or cervical change [6, 7]. Antenatal corticosteroids should be considered according to gestational age when preterm birth within 7 days is considered likely; they are not indicated solely because nonobstetric surgery is being performed [5, 7, 20]. In this patient, fetal assessment remained reassuring, no uterine activity or cervical change occurred, and neither tocolysis nor corticosteroids were administered. Antibiotic prophylaxis and mechanical thromboprophylaxis were provided; thromboembolic risk assessment is particularly important because both pregnancy and surgery increase the risk of venous thromboembolism [7, 21].

The published cases show substantial heterogeneity in clinical context and operative management (Table 1). Procedures have ranged from detorsion alone or detorsion with aspiration or cystectomy to oophorectomy, salpingo‐oophorectomy, or adnexectomy when ovarian preservation was not feasible. Most authors modified abdominal entry and port placement according to uterine size, and several used reduced‐pressure pneumoperitoneum or gasless single‐site techniques; however, operative approaches and insufflation pressures were not uniform [8, 9, 10, 11, 12, 13, 14, 15, 16, 17, 18]. Reported maternal and neonatal outcomes were generally favorable, although the evidence is limited by small numbers, publication bias, and incomplete reporting of long‐term ovarian function.

**TABLE 1:** Published reports of laparoscopic management of adnexal or ovarian torsion in advanced pregnancy and associated obstetric outcomes.

Study	GA	Patient/lesion	Entry/CO_2_	Procedure/pathology	Perioperative obstetric medication	Pregnancy and neonatal outcome
Shalowitz et al. 2013 [8]	29 weeks	30 years, G3P1; left ovarian torsion; 49 × 22 × 26 mm	NR	Laparoscopic salpingo‐oophorectomy; serous cystadenoma	NR	Term vaginal delivery
Bouet et al. 2013 [9]	Third trimester; exact GA NR	Recurrent adnexal torsion during pregnancy	Laparoscopic approach; technical details NR	Conservative laparoscopic management with adnexal preservation	NR	Favorable pregnancy outcome; detailed delivery data NR
Bouquet de Jolinière et al. 2017 [10]	30 weeks	27 years, G1; twin pregnancy after ovarian stimulation; right mass 80 × 50 mm	Veress entry below xiphoid; 3 ports; 20 mmHg	Right adnexectomy; hemorrhagic necrosis with follicles and corpus luteum	No tocolysis	Pregnancy preserved; detailed delivery outcome NR
Uyanikoglu et al. 2019 [11]	30 weeks	37 years, G3P2; left torsion; ovary 134 × 100 mm	Lee–Huang entry; 3 ports; 10 mmHg	Laparoscopic oophorectomy; necrotic ovarian tissue; specimen removed through Pfannenstiel incision	17‐hydroxyprogesterone caproate after surgery	Cesarean delivery at 37 weeks; healthy infant
Cohen et al. 2020 [12]	27–38 weeks	Case series of 12 women; 4 adnexal operations	Laparoscopic; no access‐related complication in the series; case‐level pressures NR	Case‐level adnexal procedures and pathology not uniformly reported in the accessible summary	NR	Generally favorable short‐term outcomes; the reported conversion in the series occurred during appendectomy
Zhan et al. 2021 [13]	35 weeks	28 years, nulliparous; right ovarian torsion; 100 × 90 × 60 mm	Laparoscopic; detailed entry data NR	Detorsion and ovarian cystectomy; benign mucinous cystadenoma	Magnesium sulfate reported	Vaginal delivery at 40 + 2 weeks; 3000 g
Elci 2022 [14]	Third trimester; exact GA NR	Torsioned adnexal mass; detailed patient and lesion data NR	Laparoscopic approach; technical details NR	Laparoscopic management; pathology NR	NR	Favorable outcome reported; detailed delivery data NR
Shrateh et al. 2023 [15]	33 weeks	26 years; left ovarian torsion; ovary 90 × 72 × 55 mm with extensive necrosis	Open entry; 3 ports; approximately 13 mmHg	Left oophorectomy/adnexectomy; necrotic benign ovarian tissue	Dexamethasone and medroxyprogesterone acetate administered	Spontaneous vaginal delivery at 39 weeks; 3650 g; healthy infant
Lapides et al. 2023 [16]	32 + 2 weeks	35 years, G2P1; right ovarian torsion; 125 × 103 × 86 mm	Open Hasson entry 7–8 cm above the uterine fundus; 3 ports; 10–12 mmHg	Detorsion and ovarian‐sparing cystectomy; mucinous cystadenoma with hemorrhagic/ischemic change	Betamethasone	Spontaneous vaginal delivery at 40 + 2 weeks; 3550 g; Apgar 6/9
Takeda and Hayashi 2023 [17]	33 + 2 weeks	39 years, G2P1; isolated right ovarian torsion associated with an ovarian cyst	Gasless transumbilical LESS	Detorsion, aspiration, and extracorporeal cystectomy; benign mucinous cystadenoma	Magnesium sulfate and ritodrine hydrochloride for postoperative contractions	Vaginal delivery at 36 + 5 weeks; 2108 g; healthy infant
Wang et al. 2025 [18]	34 + 5 weeks	Adnexal torsion; detailed patient and lesion data NR	Laparoendoscopic single‐site surgery	LESS management; detailed procedure and pathology NR	NR	Detailed obstetric outcome NR in the short report

Abbreviations: GA, gestational age at surgery; LESS, laparoendoscopic single‐site surgery; NR, not reported.

Although ovarian‐sparing laparoscopic management has previously been reported in the third trimester, the present case adds a particularly well‐documented example to the limited literature. Torsion developed only 10 days after magnetic resonance imaging had demonstrated preserved ovarian perfusion; the adnexa was twisted approximately 720°; improvement in ovarian color and turgor was observed within 5 min of detorsion; and simultaneous ovarian‐sparing cystectomy was completed for a mature cystic teratoma at 30 + 1 weeks. In addition, postpartum ultrasonography documented preserved ovarian morphology and vascularization. Nevertheless, the single‐case design limits generalizability and does not permit conclusions regarding comparative safety or the optimal operative approach. Management should remain individualized according to maternal stability, gestational age, intraoperative adnexal findings, and local surgical and obstetric expertise.

## Author Contributions

**Aysel Nalçakan Ergünay:** conceptualization, data curation, formal analysis, investigation, methodology, writing – original draft, writing – review and editing. **Edis Kahraman:** data curation, formal analysis, investigation, writing – review and editing. **Emine Karabük:** conceptualization, methodology, supervision, writing – review and editing. **Turgut Aydın:** conceptualization, methodology, supervision, writing – review and editing.

## Funding

The authors have nothing to report.

## Ethics Statement

The authors have nothing to report.

## Consent

Written informed consent was obtained from the patient for publication of this case report and any accompanying images.

## Conflicts of Interest

The authors declare no conflicts of interest.

## Data Availability

Data sharing is not applicable to this article as no datasets were generated or analyzed beyond the clinical information presented in this case report.
